# VSD device closure in situs inversus with dextrocardia: technical challenges and solutions: a case report

**DOI:** 10.1186/s43044-025-00665-9

**Published:** 2025-07-02

**Authors:** Abhimanyu Uppal, Bhushan Shah, Rambabu Sharma, Ashok Garg

**Affiliations:** 1 Priyanka Hospital and cardiac center, Jaipur, India; 2 AIIMS Bhopal, Bhopal, India; 3 JK Lon hospital, Jaipur, India; 4 MD General Medicine, Priyanka Hospital and cardiac center, Jaipur, India

**Keywords:** Situs inversus with dextrocardia, Mirror-image dextrocardia, Ventricular septal defect, Device closure, Retrograde VSD closure, Case report

## Abstract

**Background:**

Situs inversus with dextrocardia (SI-DC) is rarely associated with congenital heart defects. Traditionally, ventricular septal defect (VSD) in such patients has been managed surgically. Percutaneous VSD device closure in SI-DC, albeit a suitable alternative, has been seldom reported. The present report describes the unique challenges of mirror-image cardiac anatomy and suggests technical modifications for successful percutaneous closure.

**Case summary:**

A 4-year-old boy, previously diagnosed with SI-DC and a moderate-size perimembranous VSD, presented with a history of poor weight gain and dyspnea. A 2D echocardiogram indicated that the septal defect was suitable for device closure. The procedure was adapted to account for the altered cardiac anatomy by adjusting the fluoroscopic angles and wire-torquing maneuvers. A Konar-multifunction occluder device MFO® 7-5mm was successfully deployed via retrograde approach without complications utilizing hemodynamic and echocardiographic guidance. No excess contrast volume or fluoroscopic radiation dose was used during the procedure due to meticulous pre-procedure planning.

**Conclusion:**

VSD device closure in atypical scenarios like SI-DC is both safe and feasible with thorough pre-procedure planning tailored to the "mirror image" cardiac anatomy.

**Supplementary Information:**

The online version contains supplementary material available at 10.1186/s43044-025-00665-9.

## Introduction

Dextrocardia is a rare congenital abnormality with an estimated incidence ranging from 1 in 8000 to 25,000 live births [1]. SI-DC associated with a perimembranous (PM) ventricular septal defect (VSD) is even rarer [2]. Most cases reported in the literature have undergone surgical closure [3, 4]. However, VSD patch closure in this scenario is challenging for surgeon due to the altered spatial orientation of cardiac structures. This requires the surgeon to improvise the approach angle, potentially increasing the risk of conduction tissue damage [5].

A percutaneous device closure approach in this scenario is ideal, as it avoids surgical scars and offers a shorter hospital stay, among other benefits. However, literature on technical modifications for successful VSD device closure in SI-DC patients, is limited [6, 7]. There is a potential for excess contrast use and radiation exposure since the interventionist faces an unfamiliar cardiac anatomy.

In the present case report, we share our experience of closing a perimembranous VSD using retrograde technique with a Konar multifunction occluder (MFO) device in the setting of SI-DC. We aim to provide insights into technical modifications for proper device deployment and use of echocardiographic and hemodynamic guidance to avoid excess contrast and radiation exposure.

## Case report

A 4-year-old boy presented with history of poor weight gain and dyspnea with easy fatiguability while playing and running. Incidental murmur at birth had led to the diagnosis of SI-DC with perimembranous VSD. Past medical history was suggestive of recurrent respiratory infections and diaphoresis during infancy for which oral diuretics were started. Patient was subsequently lost to follow-up before presenting to our institute with aforementioned complaints.

On examination, the patient had a blood pressure of 94/60 mmHg in the right upper limb with a pulse rate of 100 beats per minute. The child was underweight for his age (16.0 kg; expected weight ≈ 18–19 kg) with a height of 98cm (within two standard deviations for his age). Cardiovascular examination revealed the apex beat in the right 5th intercostal space at mid-clavicular line with a harsh grade 4 pansystolic murmur, best heard at the lower right sternal border.

Except for mild anemia (Hb level of 10.1 gm/dL), all other relevant blood investigations were within normal range.

**Fig. 1:**
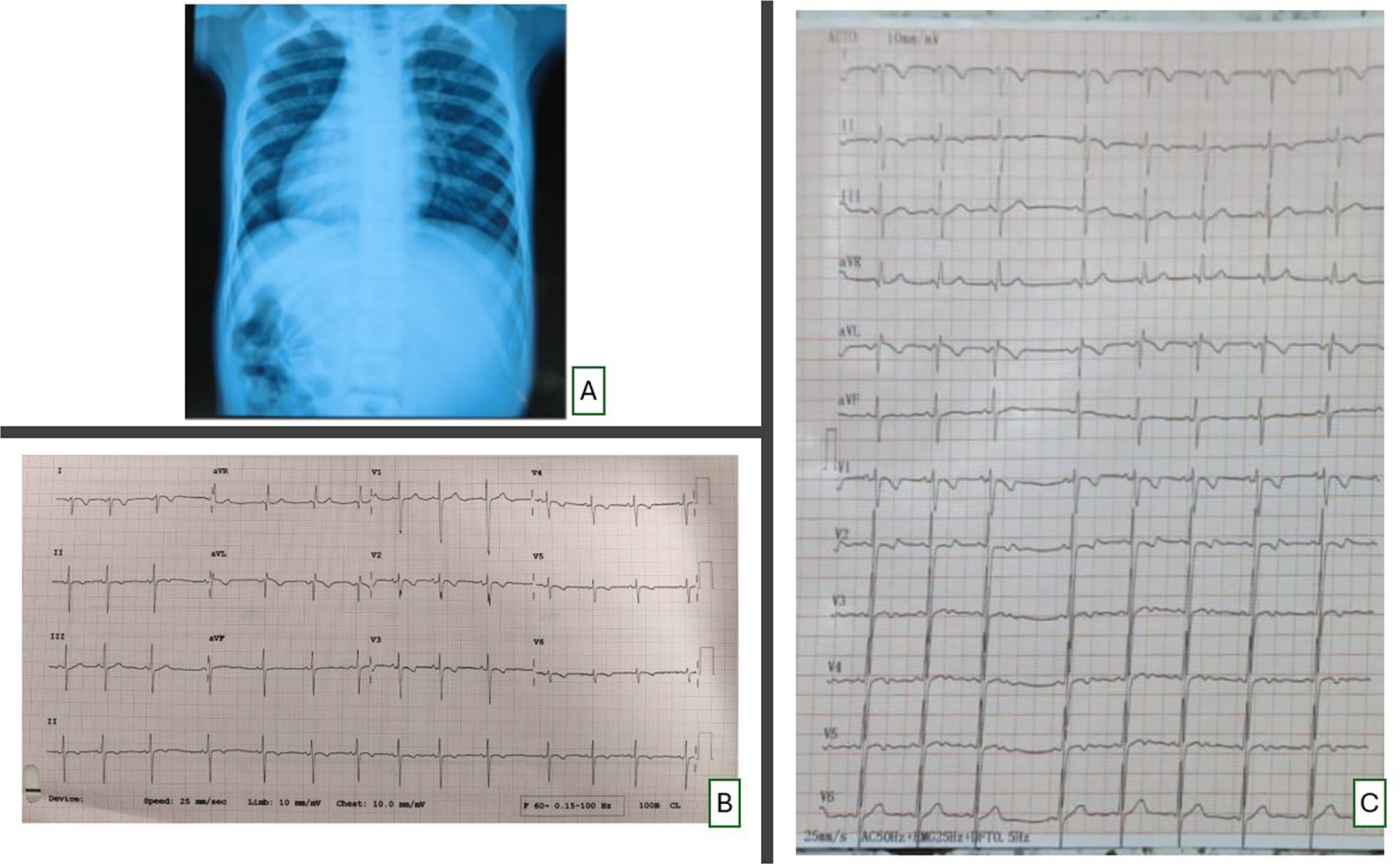
Chest X-ray and 12-lead ECG of the patient. A: Chest X-ray postero-anterior view showing mirror-image dextrocardia with cardiomegaly and prominent pulmonary arterial markings. B: Twelve lead electrocardiogram of the patient with leads placed in conventional locations. Inverted P and T waves in leads I and aVL, upright P, QRS, and T waves in lead aVR, and progressively decreasing R wave amplitude from V1 to V6 all suggest dextrocardia with situs inversus. C. Twelve lead electrocardiogram of the patient with precordial leads positioned in mirror image positions on right side of chest. Normal R wave progression is seen from V1 to V6

A chest X-ray indicated mirror-image dextrocardia with cardiomegaly (cardiothoracic ratio = 0.67), with notable bilateral pulmonary plethora (Fig. 1A).

A conventional 12-lead ECG showed inverted P and T waves in leads I and aVL, upright P, QRS, and T waves in lead aVR, and progressively decreasing R wave amplitude from V1 to V6 (Fig. 1B). Normal R wave progression was noted when precordial leads were repositioned in their corresponding mirror-image locations on the right side of chest (Fig. 1C).

**Fig. 2:**
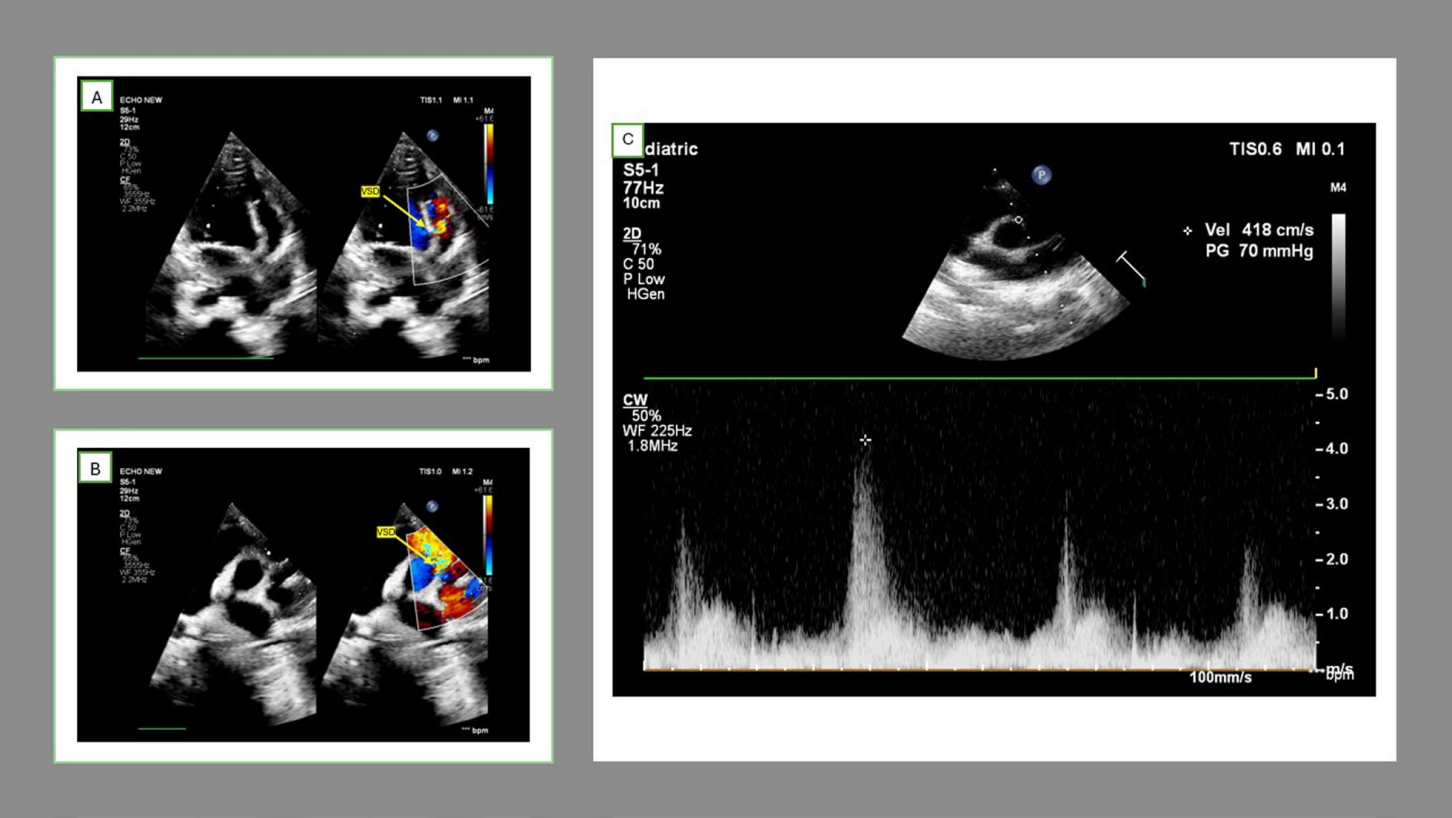
Echocardiogram of the patient showing tunnel shaped perimembranous VSD. **A** four chamber view showing color flow turbulence across the defect. **B** Parasternal short axis view showing VSD jet at 2’ o clock location. **C** Pressure gradient across VSD noted in parasternal short axis view. *Abbreviations* LV, left ventricle, RV, right ventricle, PA, Pulmonary artery, VSD, ventricular septal defect

Two-dimensional echocardiography (2D ECHO) with color Doppler confirmed situs inversus with dextrocardia, a dilated left atrium, and left ventricle (left ventricle end diastolic diameter, LVEDD = 40.5 mm, z-score = + 2.17) with a PM-VSD (3 mm toward the right ventricle, RV exit and 5 mm at the left ventricle, LV entry site) with a left-to-right shunt. This shunt was best visualized in the right parasternal short-axis view at the 2 o'clock position (mirror image of the usual 10 o’clock location for PM-VSDs) (Fig. 2A, B). The VSD was restricted by septal tricuspid adhesions and a peak gradient of 70 mmHg was noted across VSD (Fig. 2C). The defect was separated from aortic valve by a distance of 3 mm.

Combined with the patient’s medical history, the final diagnosis was SI-DC with PM VSD with significant left to right shunt that needed closure.

After obtaining informed consent from parents, patient was considered for closure. The procedure was performed under local anesthesia and conscious sedation. Cardiac catheterization suggested a significant left-to-right shunt with a suitable pulmonary vascular resistance/systemic vascular resistance (PVR/SVR) ratio (Table 1).

**Table 1:** Catheterization data. Hemodynamic recordings and oximetry readings of various cardiac chambers

	Pressure (mmHg)	Oximetry (% saturation)
Right atrium mean	6	75.5
Right ventricular systolic pressure	39	88
Pulmonary artery	40/15/21	90
Pulmonary capillary wedge pressure (mean)	7	100
Left ventricular systolic pressure	110	100
Aorta	108/70/84	99
QP/QS = 2.35, Pulmonary vascular resistance to systemic vascular resistance ratio = 0.09		

Following catheterization study, VSD device closure was performed in the same sitting via transfemoral arterial access with plan to proceed via retrograde device deployment technique.

**Fig. 3:**
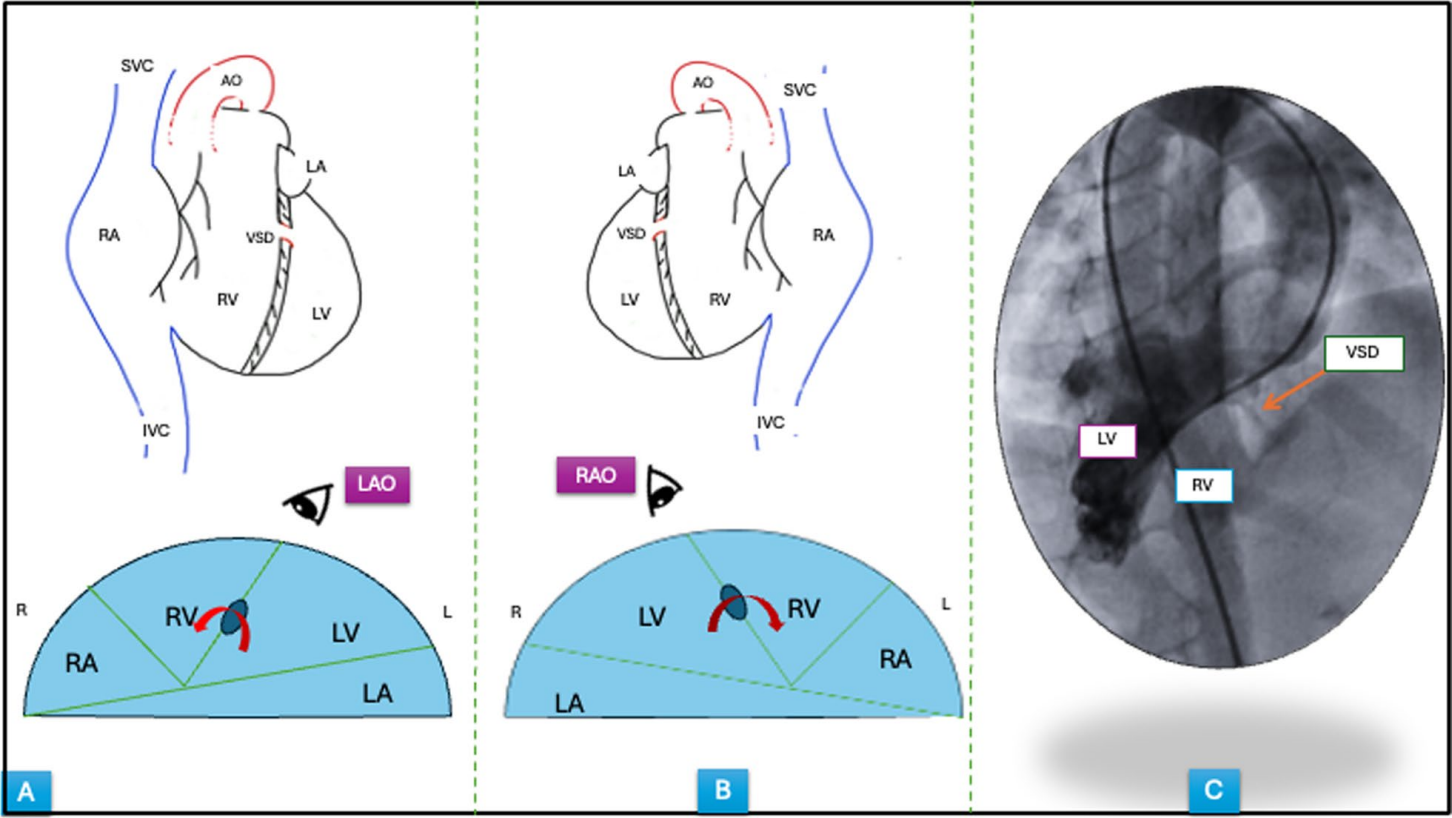
Schematic drawings of normal vs. mirror image anatomy and LV angiogram. Normal heart, **A** and situs inversus, dextrocardia heart, **B** Upper panels show the reversed right to left relationship of cardiac structure in mirror-image anatomy, and lower panels show preserved anterior–posterior relationship. The best fluoroscopic angles to visualize the defect in interventricular septum are shown in lower panels. **C** LV angiogram done for the patient in RAO with cranial projection (40°–20°). *Abbreviations* SVC, superior vena cava, IVC, inferior vena cava, LV, left ventricle, RV, right ventricle, LA, left atrium, RA, right atrium, VSD, ventricular septal defect, PA, pulmonary artery, Ao, aorta, LAO, left anterior oblique, RAO, right anterior oblique

In SI-DC, the left–right relationship of cardiac structures is reversed while the anterior–posterior relations remain unchanged. The base to apex axis of interventricular septum points from posterior to anterior in a rightward direction [4]. Therefore, the septal defect would be best profiled in a right anterior oblique view (opposite to the conventional left anterior oblique view) while maintaining the usual cranial angulation (Fig. 3A–C).

**Fig. 4:**
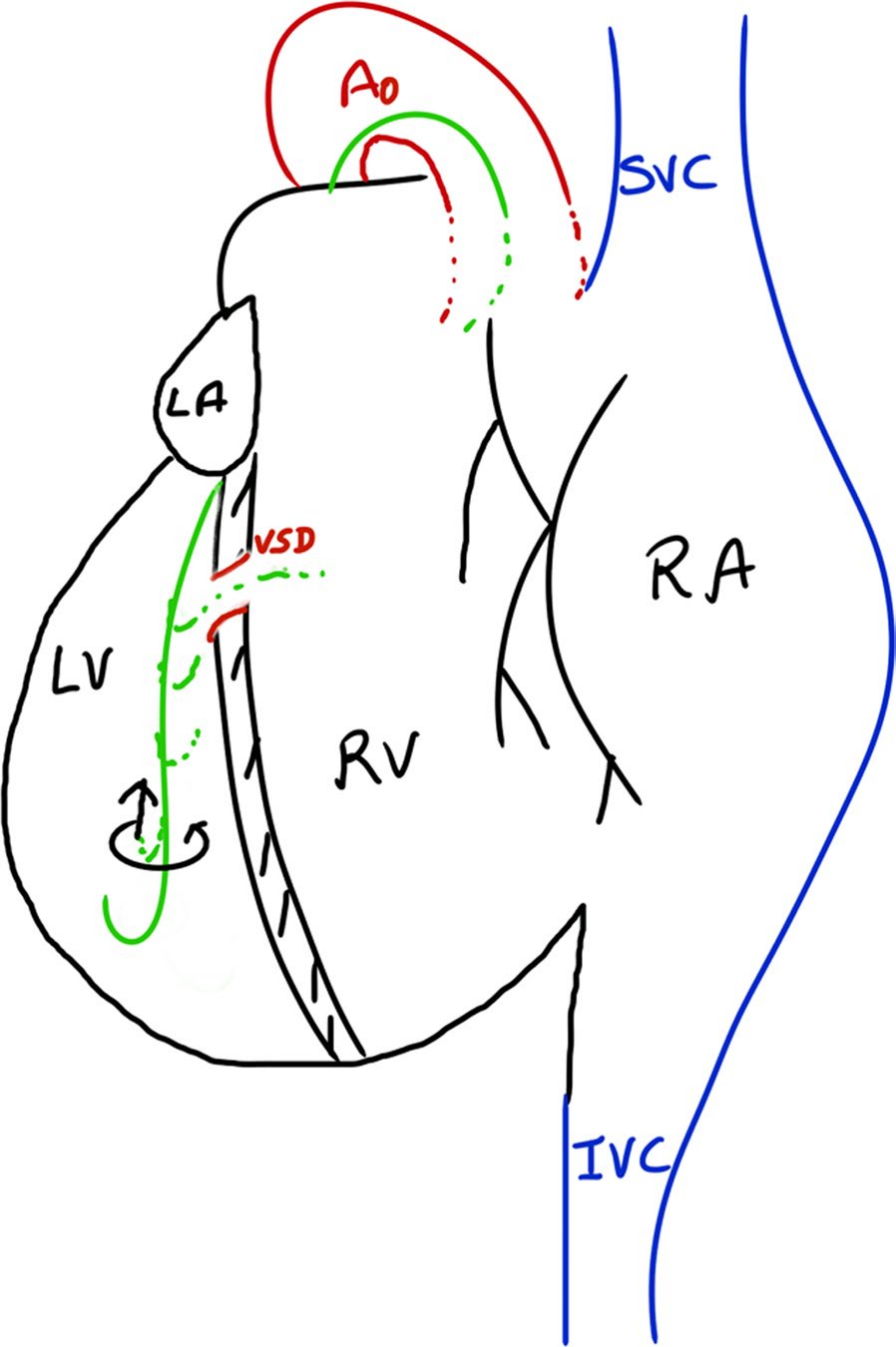
Modified wire-torquing Schematic drawing of situ inversus with dextrocardia heart showing use of counter-clockwise torque (arrows) during withdrawal of guidewire (green) to engage and cross the VSD. *Abbreviation* VSD, ventricular septal defect

To facilitate crossing the VSD from the LV side in SI-DC, we had to modify our technique of wire crossing by making a counterclockwise torque (instead of clockwise torquing) such that the wire faced the leftward-located RV and crossed the VSD (Fig. 4).

With this pre-procedure planning, a 5F pigtail catheter was advanced through right femoral arterial access and an LV angiogram was performed in RAO-CRAN 40°–20° projection to delineate the VSD. The defect was then crossed with a Terumo 0.035 (260 cm) wire which was then stationed into a branch of the left pulmonary artery. The remaining steps were the same as standard retrograde VSD closure procedure.

Pigtail catheter was exchanged for 5F Lifetech Steerease®the delivery sheath. A Konar MFO device 7-5 mm (2 mm larger than the smallest diameter measured on echo) was mounted on a 3F delivery cable and advanced through delivery system.

**Fig. 5:**
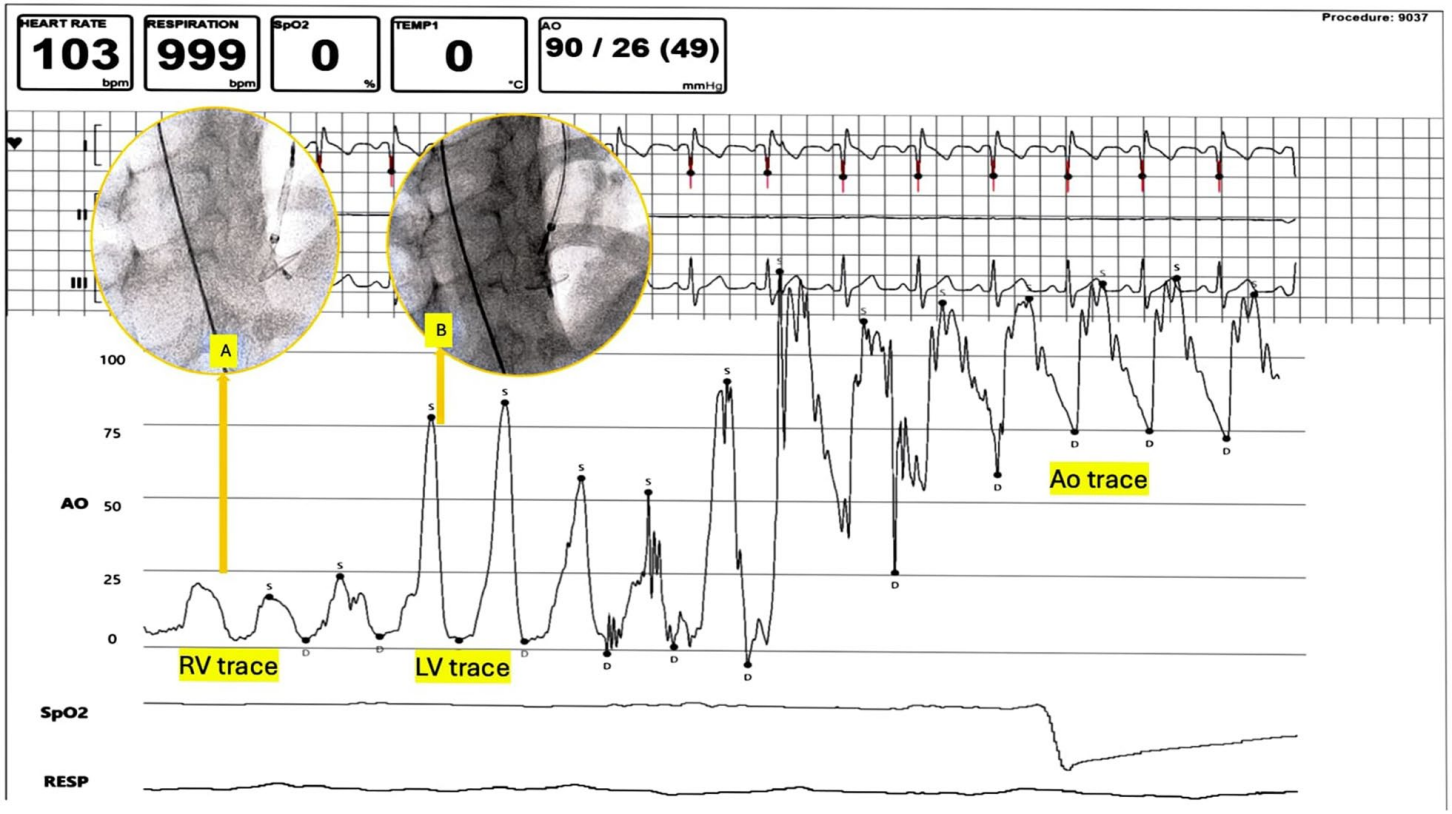
VSD deployment under hemodynamic guidance. The right disk of MFO device is unsheathed in RV cavity (inset **A**) and the entire delivery system is withdrawn with close monitoring of pressure tracings. As soon as LV tracing is noted, the entire device is deployed (inset **B**). *Abbreviations* RV, right ventricle, LV, left ventricle, Ao, aorta, VSD, ventricular septal defect, MFO, multifunction occluder

The device deployment steps were done under hemodynamic and echocardiographic guidance. Since MFO is a very low-profile device, continuous hemodynamic pressure recordings can be obtained through side-arm of delivery system even when the device is slenderized inside the sheath. The low-pressure disk of the device was unsheathed in the RV cavity, while the delivery sheath was connected to the pressure tracing monitor. At this point, RV pressure tracings were noted on the monitor. The sheath-device assembly were withdrawn together, and when the pressure tracings were noted to transition to LV pressures, the remaining part of the device was unsheathed (Fig. 5). The optimal device position was reconfirmed by bedside echo. Post-device release, LV angiogram confirmed optimal device position (Fig. 5, Video).

The total contrast volume used was 35 ml (upper limit of contrast defined by creatinine clearance for this patient was 95 ml). The fluoroscopic time, air kerma, and DAP for this procedure were 6.4 min (average: 5.5–8.5 min for this procedure at our institute), 172 mGy (average: 161-250mGy) and 8.70 Gy-cm^2^ (average: 7.5–9.5 mGy-cm^2^). The procedure was completed in 25 minutes without any intra-procedural complications.

The patient had an uneventful recovery and was discharged after 24 h of observation. He continues to be in our regular outpatient follow-up for last 6 months. The patient had improvement in dyspnea post-intervention and has gained 3.8 kg weight in the last 6 months. Serial ECHO examinations suggested that the VSD device has remained in situ, without any residual shunt or aortic/tricuspid valvulopathy. No conduction system disturbances were noted over the last six months of follow-up.

## Discussion

SI-DC complicated by VSD is a rare clinical scenario conventionally treated by open surgery [3, 4]. This cardiac malposition may pose difficulties for the surgeon and carries a potential risk of complications like conduction system damage. [5]

A percutaneous approach, while ideal, requires careful pre-procedure planning to avoid multiple contrast injections and excess radiation exposure when attempting to close the defect in an unorthodox cardiac position. Device deployment steps must be tailored to the unfamiliar cardiac anatomy.

As described earlier, in SI-DC the base-to-apex axis of the interventricular septum faces rightward in the chest cavity, with a reversed right-left relationship of cardiac structures. Technical modifications required include: (1) changing the fluoroscopic projection to RAO-CRAN for the LV angiogram, (2) applying counterclockwise torque (instead of the usual clockwise) to advance the guidewire across the VSD into the left-oriented RV, and (3) using hemodynamic and echocardiographic assistance during device deployment. The procedure time can be further reduced by choosing the retrograde technique of device deployment. Previous reports of device closure in this scenario have used conventional arteriovenous (AV) loop formation method [6] or relied on contrast injections for device positioning. [7] Conventional AV loop method can be challenging in SI-DC anatomy as described previously [6]. Device deployment under hemodynamic and echocardiographic guidance can obviate the need for excess contrast and radiation use as described in present case.

For small- to moderate-sized VSDs, our institutional protocol is to use Amplatzer duct occluder II and Konar MFO devices. In this case, we used the Konar MFO device- a self-expandable, double-disk device made from a layer of nitinol wire mesh. The device has a 2.4-mm-long hub with a screw attached to retention disks on either side, allowing it to be deployed retrogradely or antegradely [8].

To the best of our knowledge, use of this device for VSD closure in SI-DC setting has not been reported yet.

## Conclusion

Device closure of VSD in atypical scenarios such as SI-DC is both safe and feasible if meticulous pre-procedural planning is undertaken with consideration of the altered cardiac anatomy. Modification of fluoroscopic angles and wire torquing maneuvers along with use of hemodynamic guidance form the crux of a safe and successful intervention in this scenario.

## Supplementary Information

Additional file 1.

Additional file 2.

## Data Availability

No datasets were generated or analysed during the current study.

## Abbreviations

SI-DC: Situs inversus with dextrocardia
PM: Perimembranous
VSD: Ventricular septal defect
MFO: Multifunction occluder
ECG: Electrocardiogram
2D ECHO: Two-dimensional echocardiography
LVEDD: Left ventricular end-diastolic diameter
RV: Right ventricle
LV: Left ventricle
IVC: Inferior vena cava
RA: Right atrium
PA: Pulmonary artery
PVR: Pulmonary vascular resistance
SVR: Systemic vascular resistance
QP/QS: Pulmonary blood flow/systemic blood flow ratio
RAO: Right anterior oblique
CRAN: Cranial (used in fluoroscopic angle)
Hb: Hemoglobin
