# Supplementary material for: VSD device closure in situs inversus with dextrocardia: technical challenges and solutions: a case report
Source: Egypt Heart J. 2025 Jul 2;77:69. doi: 10.1186/s43044-025-00665-9 (PMC12222596; doi:10.1186/s43044-025-00665-9)
Supplement: Supplementary file 1 — Additional file 1. [file 43044_2025_665_MOESM1_ESM.docx]

**CARE Checklist of information to include when writing a case report**

| **Topic** |  | **Item** | **Checklist item description** |  |  | **Reported on Line** |
| --- | --- | --- | --- | --- | --- | --- |

**Title 1** The diagnosis or intervention of primary focus followed by the words “case report” . . . . . Page 1, Line 1,2. . . . . . . . . . . . . . .

| **Clinical Findings**  **Timeline**  **Diagnostic**  **Assessment**  **Therapeutic Intervention**  **Follow-up and Outcomes**  **Discussion**  **Patient Perspective**  **Informed Consent** |
| --- |

**Key Words 2** 2 to 5 key words that identify diagnoses or interventions in this case report, including "case report" **Pg. 1, Line 26,27**

**Abstract 3a** Introduction: What is unique about this case and what does it add to the scientific literature? . . .**Pg 1, Line 4-10.**

**(no references) 3b** Main symptoms and/or important clinical findings …**Page 1, Lines 11-15**

**3c** The main diagnoses, therapeutic interventions, and outcomes . . . . **Page 1, Lines 16-21**.

**3d** Conclusion—What is the main “take-away” lesson(s) from this case? . **Page 1, Lines 22-25**

**Introduction 4** One or two paragraphs summarizing why this case is unique (**may include** reference**s**) . **Page 2, Lines 30-48**

**Patient Information 5a** De-identified patient specific information. . . . . . **Page 2, Lines 52,53**

**5b** Primary concerns and symptoms of the patient. . . . . . . . **Page 2,3, Lines 54-64.**

**5c** Medical, family, and psycho-social history including relevant genetic information . . **Page 3, Lines 65-67**. . .

**5d** Relevant past interventions with outcomes . . . . . N/A . . . . . . . . . . . . . . . . . . . . . . . . . . . . . . . . . . . . .

1. Describe significant physical examination (PE) and important clinical findings. . . . **Page 3, Lines 70-78**
2.
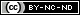

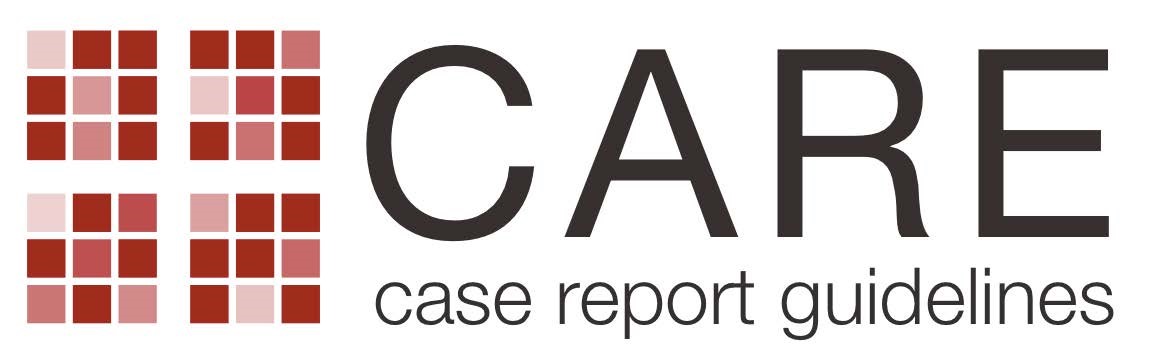
Historical and current information from this episode of care organized as a timeline . . .N/A . . . . . . . . . . . .
3. **8a** Diagnostic testing (such as PE, laboratory testing, imaging, surveys). . . . . . **Page 3,4, Lines 79-106.** . . .

**8b** Diagnostic challenges (such as access to testing, financial, or cultural) . . . . . N/A . . . . . . . .

**8c** Diagnosis (including other diagnoses considered) . . . . . . . . . . **Page 5, Lines 113-115.**

**8d** Prognosis (such as staging in oncology) where applicable . . . . . . . . N/A . . . . . . . . . .

**9a** Types of therapeutic intervention (such as pharmacologic, surgical, preventive, self-care) . **Page 5-7, Lines 118-161.**

**9b** Administration of therapeutic intervention (such as dosage, strength, duration) . **Page 5-7, Lines 118-161**

**9c** Changes in therapeutic intervention (with rationale) . . . . . . N/A . . . . . . . . . . . . . . . . . . . . . . . . . . . . .

**10a** Clinician and patient-assessed outcomes (if available) . . . . **Page 7, Lines 164-170.**

**10b** Important follow-up diagnostic and other test results . . . . . . **Page 7, Lines 164-170.**

**10c** Intervention adherence and tolerability (How was this assessed?) . . . N/A

**10d** Adverse and unanticipated events . . . . . . . . N//A

**11a** A scientific discussion of the strengths AND limitations associated with this case report. **Page 7,8 Lines 173-208.**

**11b** Discussion of the relevant medical literature **with references**. . . **Page 7, Lines 173-208.**

**11c** The scientific rationale for any conclusions (including assessment of possible causes) .Page 7,8 Lines 173-208.

**11d** The primary “take-away” lessons of this case report (without references) in a one paragraph conclusion. Page 8,Lines 211-216

1. The patient should share their perspective in one to two paragraphs on the treatment(s) they received . . N/A
2. Did the patient give informed consent? Please provide if requested . . . . . . . . . . . . . . . . . . . . . . . . . . . . . . . . . . . . . .  **Yes**
